# Vision of the active limb impairs bimanual motor tracking in young and older adults

**DOI:** 10.3389/fnagi.2014.00320

**Published:** 2014-11-17

**Authors:** Matthieu P. Boisgontier, Florian Van Halewyck, Sharissa H. A. Corporaal, Lina Willacker, Veerle Van Den Bergh, Iseult A. M. Beets, Oron Levin, Stephan P. Swinnen

**Affiliations:** ^1^Movement Control and Neuroplasticity Research Group, Biomedical Sciences Group, Department of KinesiologyKU Leuven, Leuven, Belgium; ^2^Leuven Research Institute for Neuroscience and DiseaseKU Leuven, Leuven, Belgium

**Keywords:** aging, attention, bimanual coordination, motor control, proprioception, vision

## Abstract

Despite the intensive investigation of bimanual coordination, it remains unclear how directing vision toward either limb influences performance, and whether this influence is affected by age. To examine these questions, we assessed the performance of young and older adults on a bimanual tracking task in which they matched motor-driven movements of their right hand (passive limb) with their left hand (active limb) according to in-phase and anti-phase patterns. Performance in six visual conditions involving central vision, and/or peripheral vision of the active and/or passive limb was compared to performance in a no vision condition. Results indicated that directing central vision to the active limb consistently impaired performance, with higher impairment in older than young adults. Conversely, directing central vision to the passive limb improved performance in young adults, but less consistently in older adults. In conditions involving central vision of one limb and peripheral vision of the other limb, similar effects were found to those for conditions involving central vision of one limb only. Peripheral vision alone resulted in similar or impaired performance compared to the no vision (NV) condition. These results indicate that the locus of visual attention is critical for bimanual motor control in young and older adults, with older adults being either more impaired or less able to benefit from a given visual condition.

## Introduction

Moving both hands in coordination is critical for activities of daily living such as preparing meals, eating, and dressing. To understand the mechanisms underlying the control of bimanual coordination, two typical coordination patterns have been extensively investigated. The in-phase coordination pattern is midline symmetric and involves simultaneous contraction of homologous muscles, whereas the anti-phase coordination pattern is midline asymmetric and involves alternate contractions of homologous muscles (Kelso, 1984; Swinnen, 2002; Swinnen and Wenderoth, 2004). In-phase pattern performance has consistently been shown to be more accurate and stable than anti-phase pattern performance (Wishart et al., 2000; Li et al., 2004; Howard et al., 2009; Bangert et al., 2010; Goble et al., 2010; Summers et al., 2010; Temprado et al., 2010; Gooijers et al., 2013). The decline in performance observed for the anti-phase pattern can be explained by interference between the different motor programs required for each hand (Heuer, 1993). This interlimb interference appears to alter with aging. Indeed, studies have demonstrated that bimanual coordination is less accurate and/or more variable in older compared to young adults (Temprado et al., 2010; Solesio-Jofre et al., 2014), particularly for the anti-phase pattern (Wishart et al., 2000; Lee et al., 2002; Bangert et al., 2010; Summers et al., 2010; Fling et al., 2011; Kiyama et al., 2014). However, this age effect appears to be more pronounced in intermittent than continuous tasks (Bangert et al., 2010; Summers et al., 2010). Specifically, Bangert et al. (2010) demonstrated a decline in performance in older compared to young adults on a tapping task with higher inter-tap interval variability, whereas the two groups performed similarly in both in-phase and anti-phase conditions of a continuous circle drawing task with similar corrected lag between hands. Moreover, Summers et al. (2010) found significantly higher variability in older compared to young adults on an intermittent circle drawing task with greater variations in cycle duration, whereas the two groups showed similar performance on a continuous circling task. Despite this intensive investigation of bimanual coordination, it remains unclear how directing visual attention toward either limb influences bimanual performance, and whether this influence is affected by age.

Visual attention is a central process that selects a location in the visual space for preferential stimulus processing (Balslev et al., 2013). Brain circuits mediating visual attention and visually guided saccades have demonstrated considerable overlap (Corbetta et al., 1998), and manipulating gaze direction has therefore been used as an indirect means to manipulate visual attention. In the domain of motor control, the effects of visual attention on bimanual coordination have been investigated in studies where participants were instructed to continuously draw circles in a symmetrical fashion (Swinnen et al., 1996), to continuously swing hand-held pendula (Amazeen et al., 1997; Riley et al., 1997), or to perform reciprocal tapping (Pellegrini et al., 2004) with both limbs moving actively. In these studies, which tested young adults (Swinnen et al., 1996; Amazeen et al., 1997; Riley et al., 1997) and children (Pellegrini et al., 2004), directing visual attention to the non-dominant limb resulted in consistently better performance than directing visual attention to the dominant limb. However, Alaerts et al. (2007) observed the opposite result in a task where the non-dominant limb actively tracked passive motions imposed on the dominant limb. Specifically, directing visual attention to the dominant (passively moved) hand improved bimanual tracking performance compared to a condition without visual feedback. This finding suggests that the effect of visual attention may not depend on handedness *per se*, but rather on whether the hand under visuo-attentional focus is moved actively or passively. As proprioception is less accurate in passive than active movements (Fuentes and Bastian, 2010), and because it benefits from gaze input (Wang et al., 2007; Balslev and Miall, 2008), focusing visual attention on the passive limb may improve proprioception of this limb, and in turn improve bimanual tracking performance. However, to our knowledge, it has not yet been determined whether focusing visual attention on the active limb also influences bimanual tracking performance, although behavioral performance and the central mechanisms involved would likely be different than those observed by Alaerts et al. (2007). Furthermore, previous research has not determined whether peripheral vision would result in effects similar to those observed with central vision. As different cortical networks are activated in reaching tasks under central and peripheral vision conditions (Prado et al., 2005), these visual conditions may differently affect bimanual coordination. Furthermore, the interaction effect of age and visual attention in a bimanual motor task remains unexplored to date, and may be altered by age-related decline in visual attention capacity (Wiegand et al., 2014).

To fill these gaps in the literature, we asked young and older participants to perform a bimanual tracking task under different visual conditions in order to manipulate visual attention allocated to the hands. We hypothesized that manipulating visual attention has an impact on bimanual performance. In the absence of vision, we assumed that attention would be either equally distributed between the two hands or directed mainly toward the movement of the passive hand, which had to be perceived and replicated with the active hand. We therefore, predicted that providing visual information and allocating additional attention to the passive limb would improve perception of the limb, and would in turn improve bimanual tracking performance (Alaerts et al., 2007). Conversely, we predicted that allocating visual attention to the active limb would withdraw attention from the reference limb and in turn impair bimanual tracking performance. We also predicted that the low visual discrimination associated with peripheral vision (To et al., 2011) would prevent performance improvement, and might interfere with proprioception. We further predicted that, due to age-related decline in visual attention capacity (Wiegand et al., 2014), older adults would be either more impaired or less able to benefit from a given visual condition.

## Materials and methods

### Participants

Thirty-five young (21.7 ± 2.5 years; 15 females) and thirty-one older (70.0 ± 5.8 years; 15 females) healthy volunteers participated in the study. All participants were right-handed according to the Edinburgh Handedness Inventory (Oldfield, 1971). The average lateralization quotient was similar between young and older adults (+91 ± 16 vs. +90 ± 19, respectively, with a +100 score representing extreme right-hand preference and a −100 score representing extreme left-hand preference). All participants had normal or corrected-to-normal vision, and none reported neurological, psychiatric, cardiovascular, or neuromuscular disorders. Older participants were screened for cognitive impairments with the Montreal Cognitive Assessment test (MoCA) using the standard cutoff score of 26 (Nasreddine et al., 2005). All participants gave their written informed consent, and procedures were performed according to guidelines established by the ethics committee for biomedical research at the KU Leuven, and in accordance with the WMA International Code of Medical Ethics (World Medical Association Inc., 1964).

### Apparatus

**Figure 1:**
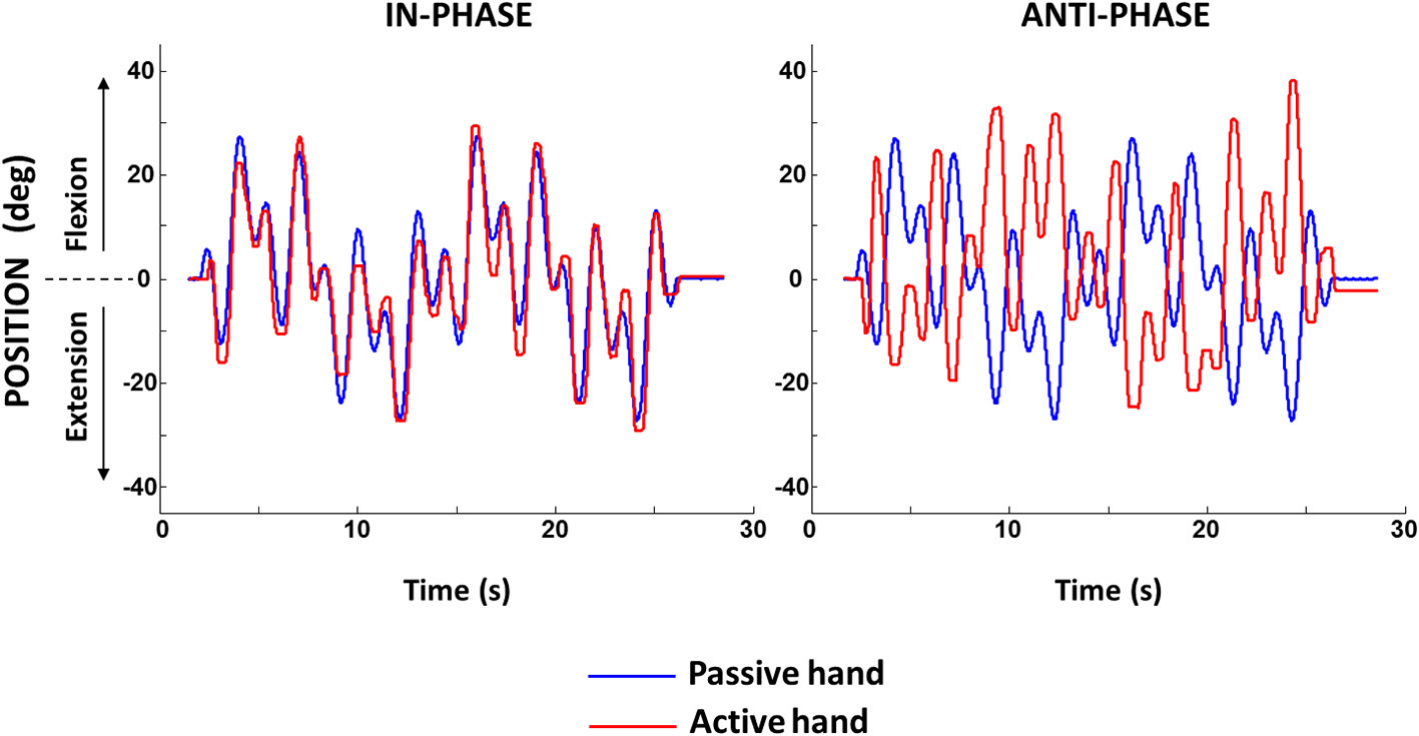
**Sample of motor-generated motion in the passive hand and tracking motion of the active hand for in-phase and anti-phase conditions**.

A custom-built apparatus was used to impose flexion-extension movements of the right wrist (passive limb; Alaerts et al., 2007). The apparatus consisted of two separate, adjustable units (left and right), both equipped with a forearm support and a manipulandum for insertion of the hand palm. Motion of the right wrist joint was induced by an AC Servo Motor (AMK DV764, Goedhard PMC, Helmond, NL) mounted underneath the right unit and coupled to the rotating shaft of the manipulandum via a 1:10 redactor (Alpha LP120 Gearbox). The motor generated a continuous but irregular sinusoidal motion with programmable amplitude, frequency, and duration to allow wrist rotation from −30° (flexion) to +30° (extension) relative to a 0° neutral position with the forearm and palmar hand surface aligned (Figure 1). Specifically, the movement consisted of a superposition of sine waves, resulting in a quasi-random movement with a mean frequency of 0.75 Hz and amplitude variations that varied between trials in a given condition to prevent prediction and anticipation. The left hand piece was constructed similarly but allowed free flexion-extension wrist movement (active limb). Shaft encoders (accuracy = 0.088°) were connected to the rotating axis to record angular displacement of the left and right wrist. Data were sampled at 1000 Hz (Signal software 4.0, Cambridge Electronic Design, Cambridge, UK) and low-pass filtered (second-order Butterworth, cut-off frequency 8 Hz, zero-lag). The angular displacement signals of the two hand pieces were stored for offline analysis. Electromyographic (EMG) activity from the right and left flexor carpi radialis and extensor carpi radialis muscles of the wrists was monitored throughout the experiment to control for the absence of muscle activity. EMG signals were amplified (×1000), filtered (4–500 Hz), sampled at 1000 Hz, and synchronized with the manipulandum signals.

### Procedures

**Figure 2:**
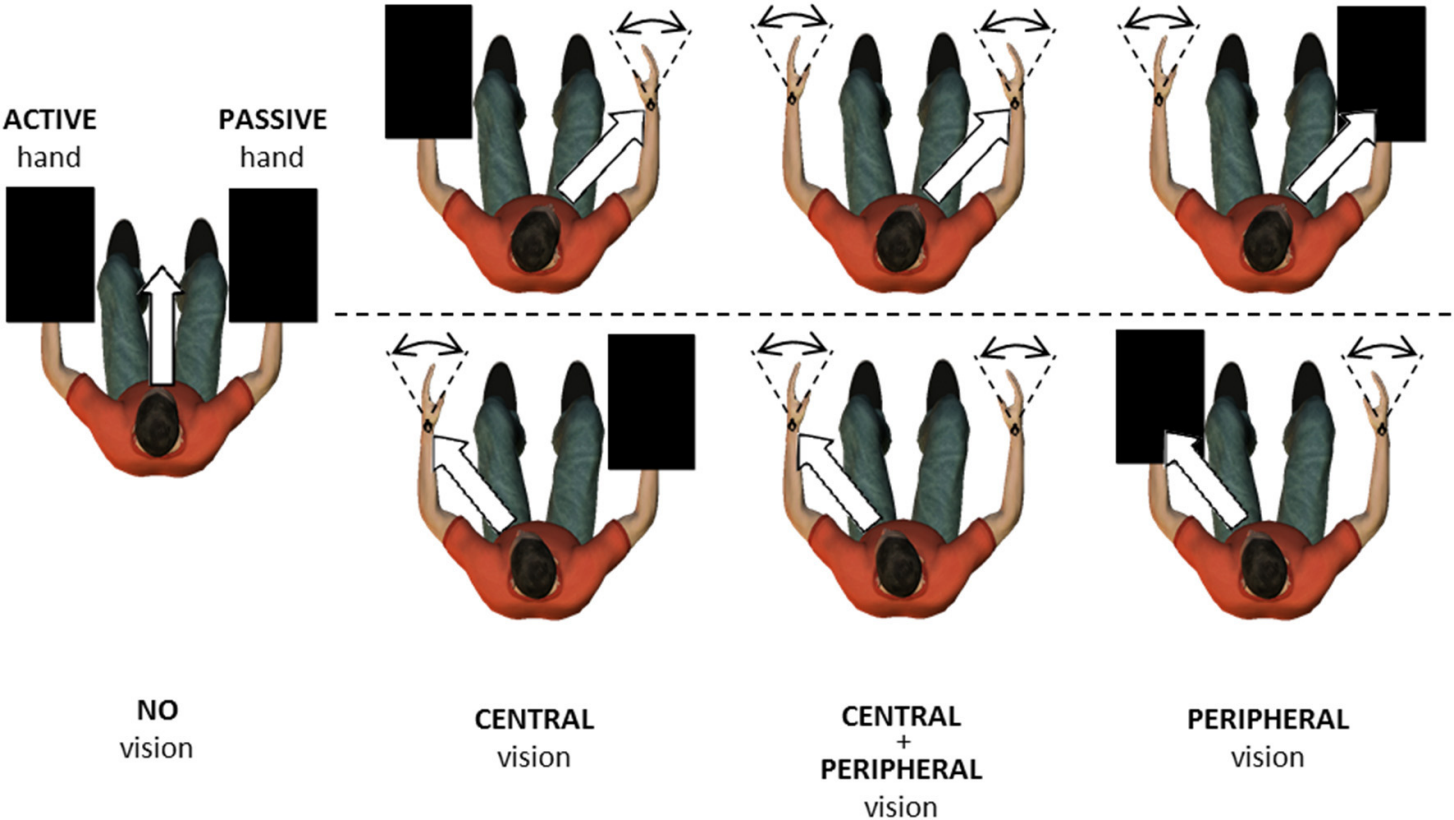
**Top view of the experimental setup in No vision, Central vision, Central + Peripheral vision, and Peripheral vision conditions**. In all conditions, participants were instructed to match a motor-driven right-hand movement (passive) with their left hand (active). Wrist movements ranged from 30° flexion to 30° extension (dashed lines). In some conditions, upper limbs were occluded by opaque boxes, here presented as black rectangles. White arrows indicate the gaze direction toward the right passive wrist (upper row) and left active wrist (lower row).

Participants were seated in front of the apparatus with shoulders in slight abduction (10–20°), elbows at 90°, and forearms supported in neutral prosupination. The Donders confrontation test was used to check participants' ability to perceive their hand movements in their peripheral field of vision while fixating on the other hand. Specifically, participants were instructed to keep their gaze fixed on one hand. The experimenter then moved his or her hand out of the participant's contralateral visual field and slowly brought it back in again. The participant was instructed to signal when the experimenter's hand came back into peripheral view. For all participants, the hand contralateral to the fixated hand was in the field of vision. In the tracking task, participants were instructed to match the motor-driven right-hand (passive) movement with their left hand (active) as accurately as possible in space and time. Tracking was performed in seven conditions (Figure 2): no vision (NV), central vision of the passive wrist (C_P_), central vision of the active wrist (C_A_), peripheral vision of the passive wrist (P_P_), peripheral vision of the active wrist (P_A_), central vision of the passive wrist and peripheral vision of the active wrist (C_P_ + P_A_), and central vision of the active wrist and peripheral vision of the passive wrist (C_A_ + P_P_). The NV condition served as a reference condition, where participants were instructed to fixate on a cross in front of them at eye level while opaque boxes prevented visual information about the upper limbs. Consequently, they could use only proprioceptive feedback to match the movement imposed by the torque motor, and visual attention was not allocated to a specific side. In the six remaining conditions, central and/or peripheral vision of the active or passive limb was added to test the effects on movement control. In both central and peripheral vision conditions, participants were instructed to extract as much information as possible. However, the central vision conditions were assumed to allow greater visual attention than the peripheral vision conditions. The combination of central and peripheral vision was used to test our hypotheses in a more ecological setting.

Testing of in-phase and anti-phase patterns was performed in the seven conditions. The two patterns were performed in distinct blocks separated by a 5-min break. Block order was counterbalanced across participants. Each block began with 3 practice trials of the tested tracking pattern (i.e., in-phase or anti-phase), followed by 28 experimental trials (7 conditions × 4 repetitions per condition) that lasted 30 s each with approximately 15 s rest between trials. Experimental trials were administered in random order. In total, each participant performed 56 recorded trials. During tracking, participants were instructed to fully relax their torque-driven right hand. When muscle activity was observed in the EMG, they were instructed to relax their wrist.

### Analysis of tracking performance

Tracking task performance in terms of time and space was assessed using the root mean square (RMS) of the phase error and amplitude error, respectively. The RMS error, also called *total variability, total error*, or simply *E*, is explained equally by the response variability and bias (RMS^2^ = Variable Error^2^ + Constant Error^2^; Henry, 1975). The RMS was therefore preferred over the absolute error, a more complex relationship between the response variability and bias that complicates the determination of the relative contribution of each component (Schutz and Roy, 1973).

#### Phase error

The relative phasing between joint angle pairs was obtained from the instantaneous phase of each signal, derived from the Hilbert transform (Boashash, 1992a,b). Relative phase was defined as the subtraction of the phase angle of the left (active) from that of the right (passive) wrist, according to the following formula (Scholz and Kelso, 1989):

$$\Phi =\theta_{RW}-\theta_{\text{LW}}=\tan^{-1}\left(\frac{{\text{dX}}_{RW}/\text{dt}}{X_{RW}}\right)-\tan^{-1}\left(\frac{{\text{dX}}_{LW}/\text{dt}}{X_{LW}}\right)$$

where θ_*RW*_ and θ_LW_ are the phase of the right and left wrist movement in each sample, respectively; X_*RW*_ and X_*LW*_ are the position of the right and left wrist after rescaling to the interval [−1,1] for each oscillation cycle; and dX_*RW*_/dt and dX_*LW*_/dt are the normalized instantaneous velocity. The RMS of the relative phase (Phase error) was then calculated according to the following formula:

$$\text{Phase error}=\{\begin{array}{l} \sqrt{\frac{1}{\text{N}}\times \sum_{1}^{\text{N}}{\left(\Phi -180\right)}^{2}}\text{ In-phase condition}\\ \sqrt{\frac{1}{\text{N}}\times \sum_{1}^{\text{N}}{\left(\Phi \right)}^{2}}\text{ Anti-phase condition} \end{array}$$

where N is the number of data samples over a trial of 24 s (2.4 × 10^4^), with the first and last 3 s of each 30-s trial removed from analysis.

#### Amplitude error

Spatial performance was derived from the continuous displacement series for each wrist, and the RMS error of the amplitude (Amplitude error) was defined according to the following formula:

$$\text{Amplitude error }=\{\begin{array}{l} \sqrt{\frac{1}{\text{N}}\times \sum_{1}^{\text{N}}{\left({\text{x}}_{\text{LW}}-\left(-{\text{x}}_{\text{RW}}\right)\right)}^{2}}\text{ In-phase condition}\\ \sqrt{\frac{1}{\text{N}}\times \sum_{1}^{\text{N}}{\left({\text{x}}_{\text{LW}}-{\text{x}}_{\text{RW}}\right)}^{2}}\text{ Anti-phase condition} \end{array}$$

where x_LW_ and x_RW_ are the angular position of the left and right wrist, respectively.

Data were processed with an in-house program using MATLAB (version R2012b, MathWorks Inc., Natick, MA, USA). The dependent variables (i.e., Phase error and Amplitude error) were calculated for each trial and averaged across the four trials for each participant in each condition.

### Statistical analyses

To test the effects of coordination pattern and visual condition on the temporal and spatial components of bimanual coordination in young and older adults, mean Phase and Amplitude error were analyzed by 2 × 2 × 7 analyses of variance (ANOVAs) with the factors Age (Young adults, Older adults), Pattern (In-phase, Anti-phase), and Vision (NV, C_A_, C_A_ + P_P_, P_A_, C_P_, C_P_ + P_A_, P_P_). Level of significance (α) was set at *p* = 0.05. *P*-values of ANOVAs were corrected for sphericity using the Greenhouse–Geisser method when Mauchly's test was significant. When ANOVAs revealed significant effects, the false discovery rate procedure was conducted to test comparisons of interest (Curran-Everett, 2000). In line with our research questions, we focused on effects between the reference condition (NV) and the other visual conditions. Partial eta squared values (η^2^_*P*_) were reported to indicate small (≥0.01), medium (≥0.06), and large (≥0.14) effect sizes (Sink and Stroh, 2006).

Complementary analyses were run to test age-related range differences between the NV condition and visual conditions. Range difference was calculated by subtracting the mean of the NV condition from the mean of each visual condition for each participant. Data were analyzed using 2 × 2 × 6 ANOVAs with the factors Age, Pattern, and Vision (C_A_, C_A_ + P_P_, P_A_, C_P_, C_P_ + P_A_, P_P_).

## Results

### Phase error

**Figure 3:**
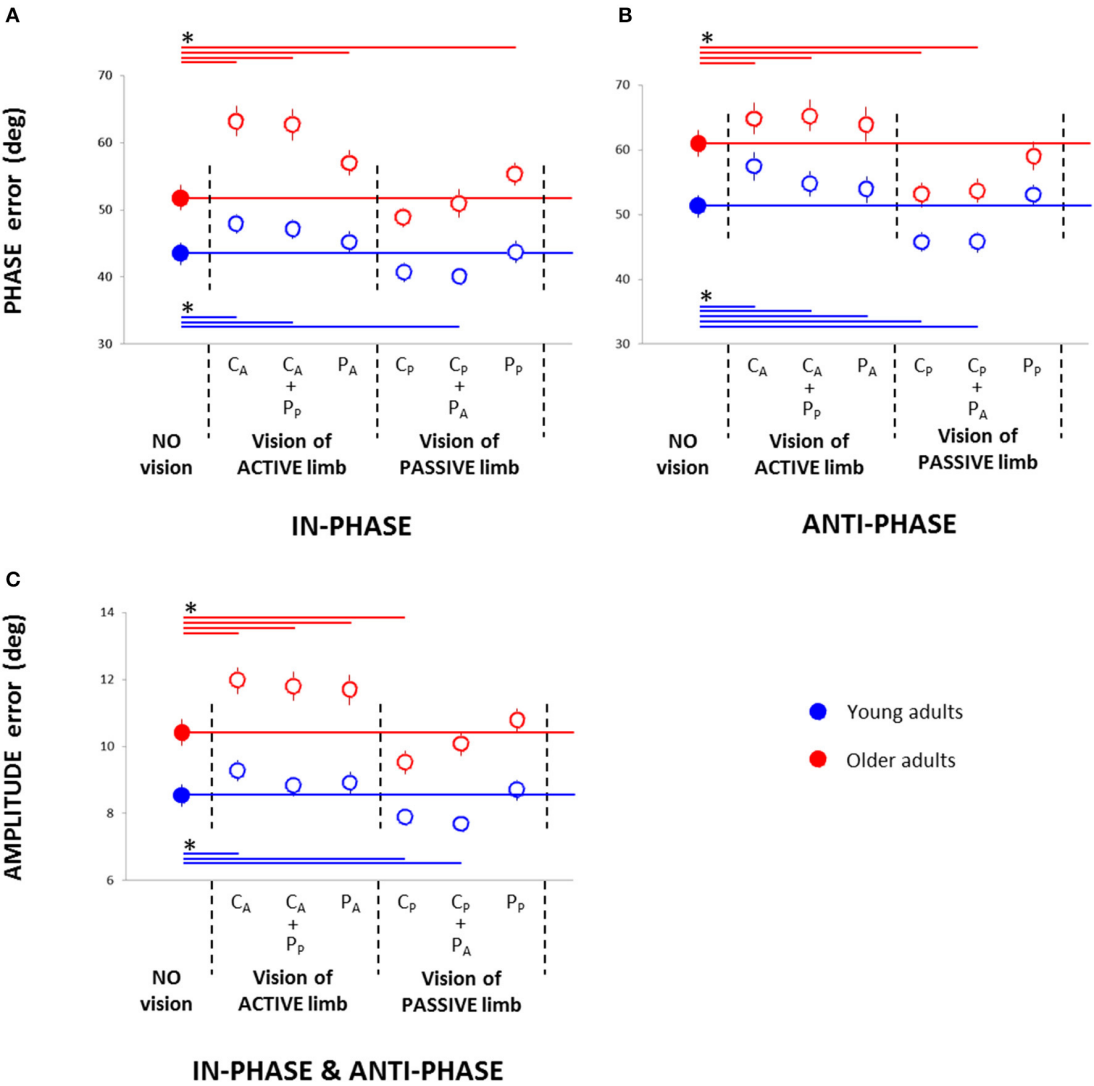
**Root mean square of the relative phase (phase error) in the in-phase (A) and anti-phase pattern (B) and root mean square of the amplitude error (C) in young and older adults in the seven conditions: no vision; central vision of the active wrist (C_A_), central vision of the active wrist and peripheral vision of the passive wrist (C_A_ + P_P_), peripheral vision of the active wrist (P_A_), central vision of the passive wrist (C_P_), central vision of the passive wrist and peripheral vision of the active wrist (C_P_ + P_A_), and peripheral vision of the passive wrist (P_P_)**. ^*^ = significant difference.

For Phase error, the Three-Way (Age × Pattern × Vision) ANOVA demonstrated significant main effects of Age [Young adults = 48 ± 2 vs. Older adults = 58 ± 2, mean ± s.e.m.; *F*_(1, 64)_ = 33.71; *p* < 0.001; η^2^_*P*_ = 0.35], Pattern [In-phase = 50 ± 2 vs. Anti-phase = 56 ± 2; *F*_(1, 64)_ = 19.56; *p* < 0.001; η^2^_*P*_ = 0.23], and Vision [*F*_(6, 384)_ = 50.02; *p* < 0.001; η^2^_*P*_ = 0.44], a significant Two-Way (Pattern × Vision) interaction [*F*_(6, 384)_ = 2.48; *p* < 0.029; η^2^_*P*_ = 0.04], and a significant Three-Way interaction [*F*_(6, 384)_ = 2.39; *p* = 0.035; η^2^_*P*_ = 0.04]. For the in-phase pattern, *post-hoc* analyses revealed that, compared to the NV condition, performance declined in C_A_ and C_A_ + P_P_ conditions for both groups (*p* < 0.021) as well as in P_A_ and P_P_ for older adults (*p* < 0.007), whereas performance improved in C_P_ + P_A_ condition for young adults (*p* = 0.029; Figure 3A). For the anti-phase pattern, *post-hoc* analyses revealed that, compared to the NV condition, performance declined in C_A_ and C_A_ + P_P_ conditions for both groups (*p* < 0.026) and in P_A_ condition for young adults (*p* = 0.036), whereas performance improved in C_P_ and C_P_ + P_A_ conditions for both groups (*p* < 0.001; Figure 3B).

### Amplitude error

For Amplitude error, the Three-Way (Age × Pattern × Vision) ANOVA demonstrated main effects of Age [Young adults = 8.5 ± 0.4 vs. Older adults = 10.9 ± 0.5; *F*_(1, 64)_ = 35.90; *p* < 0.001; η^2^_*P*_ = 0.36], Pattern [In-phase = 9.2 ± 0.4 vs. Anti-phase = 10.1 ± 0.5; *F*_(1, 64)_ = 7.43; *p* = 0.008; η^2^_*P*_ = 0.10], and Vision [*F*_(6, 384)_ = 27.82; *p* < 0.001; η^2^_*P*_ = 0.30], and a significant Two-Way (Age × Vision) interaction [*F*_(6, 384)_ = 3.40; *p* = 0.005; η^2^_*P*_ = 0.05] (Figure 3C). No other significant interaction effects were found (all *p* > 0.20). *Post-hoc* analyses revealed that, compared to the NV condition, performance declined in C_A_ condition for both age groups (*p* < 0.007) and in C_A_ + P_P_ and P_A_ conditions for older adults (*p* < 0.002), whereas performance improved in C_P_ condition for both groups (*p* < 0.024) and in C_P_ + P_A_ conditions for young adults (*p* < 0.001).

### Age-related range differences

**Figure 4:**
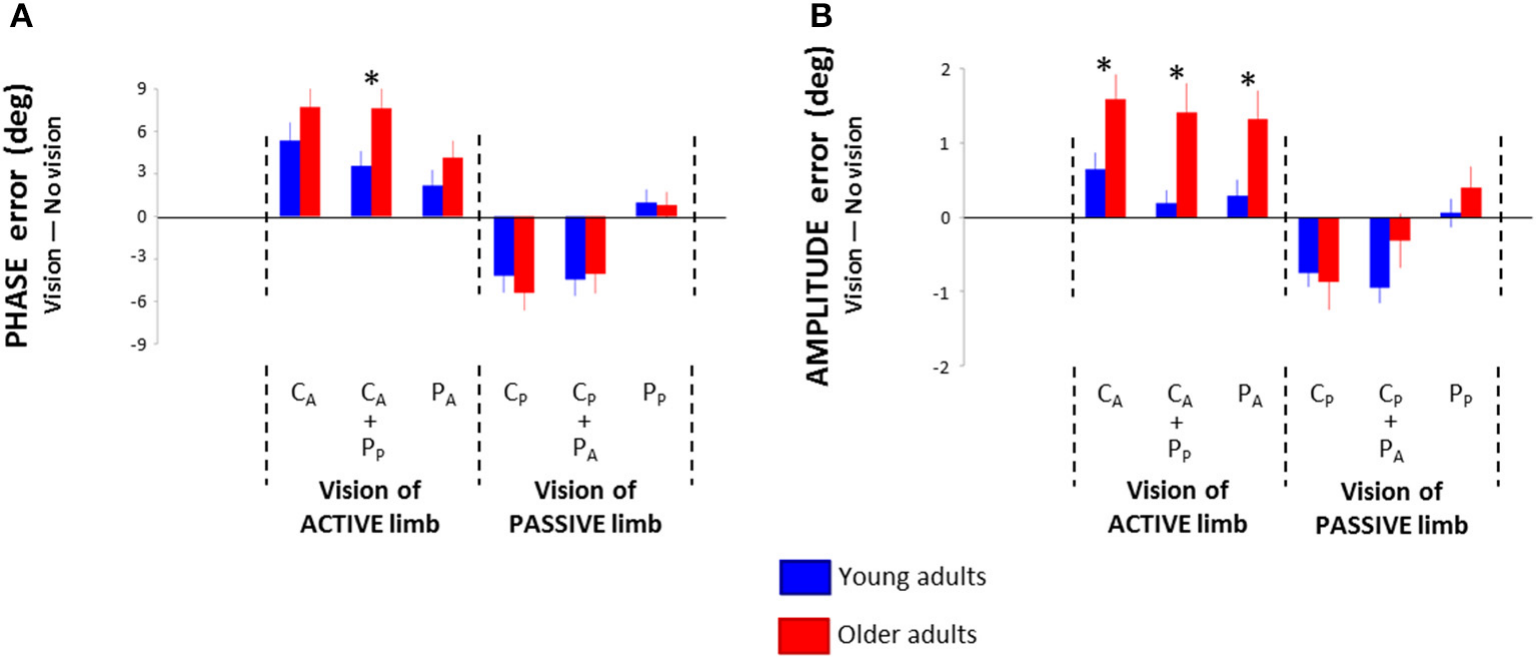
**Phase (A) and amplitude (B) root mean square difference between the no vision condition and the six visual conditions in young and older adults: central vision of the active wrist (C_A_), central vision of the active wrist and peripheral vision of the passive wrist (C_A_ + P_P_), peripheral vision of the active wrist (P_A_), central vision of the passive wrist (C_P_), central vision of the passive wrist and peripheral vision of the active wrist (C_P_ + P_A_), and peripheral vision of the passive wrist (P_P_)**. ^*^ = significant difference.

As the effects of the different visual conditions showed similar directions across age groups, we investigated age-related range differences between the NV condition and all visual conditions. For Phase error, the Three-Way (Age × Pattern × Vision) interaction of the 2 × 2 × 6 ANOVA was not significant [*F*_(5, 320)_ = 1.56; *p* = 0.178; η^2^_*P*_ = 0.02]. However, the Two-Way interaction (Age × Vision) showed marginal significance [*F*_(5, 320)_ = 2.18; *p* = 0.066; η^2^_*P*_ = 0.03]. *Post-hoc* analyses revealed significantly higher impairment in older than young adults in C_A_ + P_P_ condition (*p* = 0.021; Figure 4A). No other significant between-group effects were found (*p* > 0.198).

For Amplitude error, the Three-Way interaction was not significant [*F*_(5, 320)_ = 1.18; *p* = 0.318; η^2^_*P*_ = 0.02]. However, the Two-Way interaction (Age × Vision) was significant [*F*_(5, 320)_ = 3.03; *p* = 0.016; η^2^_*P*_ = 0.05]. *Post-hoc* analyses revealed significantly higher impairment in older than young adults in conditions involving visual attention directed toward the active limb (C_A_, C_A_ + P_P_, P_A_; *p* < 0.018; Figure 4B). Furthermore, older adults did not benefit more than young adults from visual attention directed toward the passive limb (C_P_, C_P_ +P_A_, P_P_; *p* > 0.11).

## Discussion

We investigated the effects of the locus of visual attention on bimanual tracking performance in young and older adults. Visual attention was manipulated using six visual conditions that were compared to a NV condition. Our main finding was that directing central vision to the active limb produced consistently impaired performance in both in-phase and anti-phase patterns in young and older adults, whereas drawing attention to the passive limb produced consistently improved performance, particularly in young adults. The results also showed that conditions involving central as well as peripheral vision demonstrated similar effects to those of the conditions involving central visual attention only. Furthermore, compared to the NV condition, peripheral visual attention alone resulted in similar or impaired performance.

### Age-related decline in a continuous bimanual tracking task across both in-phase and anti-phase patterns

In line with the literature, both the phase and amplitude variables showed higher error in the anti-phase compared to in-phase pattern (Wishart et al., 2000; Li et al., 2004; Howard et al., 2009; Bangert et al., 2010; Goble et al., 2010; Summers et al., 2010; Temprado et al., 2010; Gooijers et al., 2013). Our study also showed a main effect of Age but no Age × Pattern interaction, indicating age-related decline in performance in a continuous bimanual coordination task for both in-phase and anti-phase patterns. The discrepancy between our results and previous work showing no age-related effects in continuous tasks (Serrien et al., 1996, 2000; Bangert et al., 2010; Summers et al., 2010) or for in-phase patterns (Wishart et al., 2000; Lee et al., 2002; Bangert et al., 2010; Summers et al., 2010; Fling et al., 2011; Kiyama et al., 2014) can be explained by the different levels of task complexity. Indeed, all previous studies used continuous movements with stable amplitudes and frequencies, whereas we used a superposition of sine waves, resulting in a quasi-random movement trajectory and amplitude variations within trials. Due to its greater complexity, our task may have been more sensitive to age-related differences in bimanual coordination.

### Directing visual attention to the active limb impairs bimanual motor tracking

We have demonstrated for the first time that directing central vision to the active hand impairs bimanual tracking performance compared to a NV condition in both young and older adults (Figure 3). As vision is considered to improve both the perception (Corbetta et al., 1998) and control of movement (Goodale, 2011), the impaired performance we observed was probably not due to the direct effect of vision on the active limb. Instead, as attention is predominantly guided by the eye during simultaneous eye and hand movements (Khan et al., 2011), directing central vision to the active limb may have removed attention from the reference passive limb and impaired the perception of this limb. Furthermore, if we consider the possibility that attention was directed mainly toward the reference passive hand in the NV condition, focusing on the active hand may have involved additional processes that could have contributed to the impaired bimanual tracking performance. Specifically, voluntarily orienting attention toward the passive hand without eye movements requires decoupling attention from the locus of fixation, shifting to the desired location, and maintaining attention at that location (Posner et al., 1984; Kelley et al., 2008).

### Directing visual attention to the passive limb improves bimanual motor tracking

Alaerts et al. (2007) demonstrated that, compared to a NV condition, gazing at the passive hand improved bimanual tracking performance in young adults for both in-phase and anti-phase patterns. In the present study, we reproduced this effect in young adults and extended it to older adults. Overall, the research suggests that proprioceptive information carried by gaze input (Wang et al., 2007; Balslev and Miall, 2008) compensates for the decline in proprioception in the passive hand (Fuentes and Bastian, 2010). This improved perception may refine the internal representation of the movement that is to be actively performed (Wolpert and Kawato, 1998), which in turn improves bimanual tracking performance. However, this improvement may also be explained by the increased attention associated with the eye saccades guided by hand movements (Corbetta et al., 1998). In fact, increased visual attention toward a movement performed by another individual has been shown to increase the cortical excitability of the motor system (Fadiga et al., 1995; Strafella and Paus, 2000) and to activate premotor areas (Iacoboni et al., 1999; Buccino et al., 2001). These effects are also likely to occur when individuals focus on their own movements, and may impact the control of these movements. However, it remains to be determined whether this improvement is explained by the addition of proprioceptive gaze input or greater attention, or a combination of the two, an issue that merits further research.

Meanwhile, results in somatosensory studies addressing this question are inconsistent. Some results have shown that attention improves tactile perception (Tipper et al., 1998; Honoré et al., 1989). Furthermore, in a task where participants had to detect tactile stimulation of the thumb, additional visual information about the thumb demonstrated no further facilitation to that of attention alone (Tipper et al., 1998). Conversely, Kennett et al. (2001) have shown better spatial resolution of touch with than without vision of the arm, whereas viewing a neutral object at the arm's location did not improved spatial resolution, ruling out attention orienting as a possible account.

### Effects of peripheral vision

In young adults, the addition of peripheral vision appeared to compensate for the spatial impairment observed when central vision was directed to the active limb (Figure 3C), and it improved temporal performance (Phase error) when associated with central vision of the passive limb for the in-phase pattern (Figure 3A). On the other hand, in older adults, adding peripheral vision of the contralateral limb when central vision was directed to the passive limb appeared to override the spatial improvement (Amplitude error) observed in the central vision condition (Figure 3C). These results demonstrated that, when gazing at one limb, adding peripheral vision of the contralateral limb resulted in similar or improved performance in young adults and similar or impaired performance in older adults. In the condition involving peripheral vision only, focusing on the active limb consistently resulted in impaired performance in older adults (Figures 3A,C), whereas young adults showed spatial impairment for the anti-phase pattern only (Figure 3B). Although directing peripheral vision to the passive limb had no effect on young adults, it produced impaired temporal performance in older adults for the in-phase pattern (Figure 3A). These findings indicate that peripheral vision of a limb resulted in similar or impaired performance compared to no-vision performance in both age groups. However, the impairment was more consistent in older than young adults.

Taken together, these results, observed in conditions involving peripheral vision, indicate that performance in both young and older adults can be impaired by peripheral vision. This effect could be explained by lower visual discrimination in peripheral than central vision (Jonas et al., 1992; To et al., 2011), which may interfere with proprioception. In terms of attention, the fact that item selection is more easily degraded by distractors in peripheral compared to central viewing (Intriligator and Cavanagh, 2001) suggests that peripheral vision is less efficient for attentional purposes, which may also account for the impairment we observed. Furthermore, our results suggest that older adults are either more impaired or less able to benefit from peripheral vision compared to young adults, which may be explained by age-related decline in the ability to integrate multiple sensory cues (Roudaia et al., 2013) and to efficiently model a movement and its associated motor commands (Boisgontier and Nougier, 2013).

### Age-related range differences between the no vision condition and visual conditions

The investigation of age-related range differences between a NV condition and visual conditions revealed generally higher impairment in older than young adults when visual attention was directed toward the active limb (Figure 4). This age-related impairment may be associated with the attentional decoupling presumed to be at play in these visual conditions (Posner et al., 1984; Kelley et al., 2008). Thus, age-related decline in visual attention capacity (Wiegand et al., 2014) together with age-related increase in proprioceptive cost (Boisgontier et al., 2012) may prevent older adults from handling the additional load associated with attention decoupling. Conversely, young and older adults showed similar improvement in task performance when visual attention was directed toward the passive limb.

## Author contributions

Experimental conception and design: Matthieu P. Boisgontier. Experimental conduct: Matthieu P. Boisgontier, Veerle Van Den Bergh, Oron Levin. Data analysis: Matthieu P. Boisgontier, Lina Willacker, Oron Levin. Manuscript preparation: Matthieu P. Boisgontier, Florian Van Halewyck, Sharissa H. A. Corporaal, Iseult A. M. Beets, Oron Levin, Stephan Patrick Swinnen. All authors approved the final version of the manuscript.

### Conflict of interest statement

The authors declare that the research was conducted in the absence of any commercial or financial relationships that could be construed as a potential conflict of interest.
